# The role of the Hippo/YAP pathway in the physiological activities and lesions of lens epithelial cells

**DOI:** 10.3389/fcell.2025.1524814

**Published:** 2025-03-24

**Authors:** Shumei Tan, Xiaodan Jiang, Ziyuan Liu, Xuemin Li

**Affiliations:** Department of Ophthalmology, Beijing Key Laboratory of Restoration of Damaged Ocular Nerve, Peking University Third Hospital, Beijing, China

**Keywords:** Hippo/YAP pathway, lens epithelial cells, crystalline lens, opthalmological diseases, cataract

## Abstract

The Hippo/YAP pathway is a signaling pathway that plays an important role in cell proliferation, survival, differentiation, cell fate determination, organ size, and tissue homeostasis. Lens epithelial cells (LECs), located on the anterior surface of the lens, are the parental cells responsible for growth and development of the transparent ocular lens. During lens development, LECs undergo a process of differentiation where they exit the cell cycle and transform into lens fiber cells (LFCs), which constitute the majority of the lens structure. YAP is involved in the proliferation and differentiation of LECs, the maintenance of nuclear morphology, cell polarity, cell apical polarity complex, and connexin morphology. The role of the ordered arrangement of LFCs has been demonstrated in several animal studies, and *Yap1* heterozygous deletion mice exhibit cataracts. The mechanism of the Hippo/YAP pathway in the physiological activities and lesions of LECs is complex, which is of great significance to understanding the development of the lens and the pathogenesis of lens-related diseases.

## 1 Introduction

**FIGURE 1:**
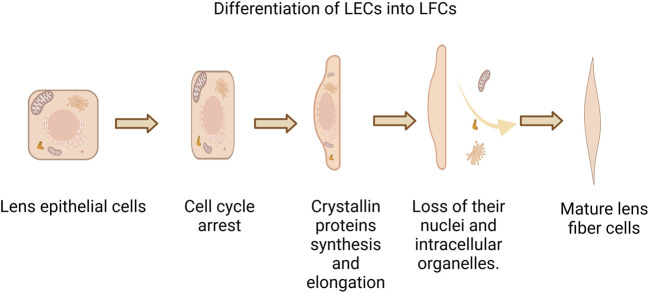
Differentiation of LECs into LFCs. During the process of lens epithelial cells (LEC) differentiating into lens fiber cells (LFC), proliferative LECs first exit the cell cycle, then synthesize crystallin proteins and elongate their cells, subsequently losing their nucleus and organelles, ultimately forming mature lens fiber cells that are devoid of nuclei and organelles.

Cataract is one of the leading causes of blindness. Lens epithelial cells (LECs) are crucial cells in the lens, continuously proliferating and differentiating into lens fiber cells (LFCs) throughout life (showed in Figure 1). The normal progression of physiological activities such as proliferation, differentiation, and apoptosis of LECs is vital for maintaining the metabolic homeostasis and transparency of the lens (Liu Z. et al., 2023). The Hippo/YAP pathway is widely present in multiple systems of higher animals, playing a significant role in cell proliferation, survival, differentiation, cell fate determination, organ size, and tissue homeostasis (Nishina, 2022). In past studies, the Hippo pathway was related to several eye conditions including lens-corneal separation defect (Wang Q. et al., 2022), cataracts (Hong et al., 2024), Uveitic glaucoma (Bitard et al., 2024), diabetic retinopathy (DR) (Zhang X. et al., 2024), age-related macular degeneration (AMD) (Yan et al., 2018), proliferative vitreoretinopathy (PVR) (Zhang and Li, 2022), retinoblastoma (RB) (Zhang et al., 2015) and uveal melanoma (UM) (Li et al., 2019). The Hippo/YAP pathway is essential for the physiological functions and pathological changes in LECs. YAP regulates lens development and pathology by modulating key processes, including the proliferation, apoptosis, differentiation, and maintenance of cellular morphology and polarity in LECs (Dawes et al., 2018; He et al., 2019; Kumar et al., 2019; Lu et al., 2020; Maddala et al., 2020; Li et al., 2023; Liu et al., 2022; Wiley et al., 2010; Deshmukh et al., 2019). This article reviews the current understanding of the role of the Hippo/YAP pathway in the physiological activities and pathologies of LECs, providing a theoretical basis for understanding the life activities of LECs and exploring treatment strategies for related diseases.

## 2 Review

### 2.1 Overview of the Hippo/YAP pathway

**FIGURE 2:**
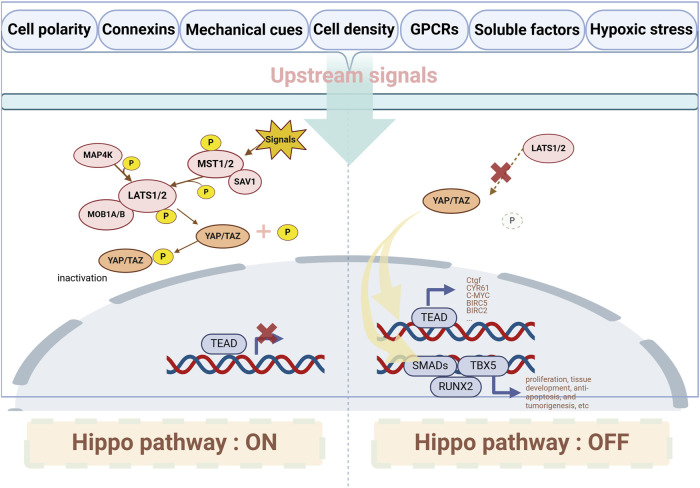
Major components and interactions of the Hippo/YAP pathway. When upstream signals activate the Hippo pathway, MST1/2 interacts with SAV1 and phosphorylates LATS1/2 to activate them; in some cases, LATS1/2 can also be activated by MAP4Ks. Once LATS1/2 is activated, it induces the phosphorylation of YAP, inhibiting its translocation into the nucleus. When the Hippo pathway is suppressed, YAP translocates into the nucleus and interacts with downstream transcriptional targets. GPCRs: G protein-coupled receptors. MST1/2: Mammalian sterile 20-like kinase 1/2; SAV1: Salvador homolog 1; LATS1/2: Large tumor suppressor 1/2; MAP4Ks: MAP kinase kinase kinase kinases; YAP: Yes-associated protein; TEAD: Transcriptional enhanced associate domain family; TEA-domain transcription factor; RUNX2: Runt-related transcription factor 2; TBX5: T-box transcription factor 5.

The main components of the Hippo pathway include MST1/2 kinases (Mammalian sterile 20-like kinase 1/2, MST1/2), Salvador homolog 1 (SAV1), MOB kinase activator 1A/B (MOB1A/B), large tumor suppressor 1/2 (LATS1/2), Yes-associated protein 1 (YAP1 or YAP), transcriptional co-activator with PDZ-binding motif (TAZ, also known as WWTR1), and the transcriptional enhanced associate domain family (TEAD) (Ma et al., 2019) (showed in Figure 2). MST1/2, SAV1, MOB1A/B, and LATS1/2 form the upstream kinase cascade of the Hippo pathway, while YAP/TAZ acts as the key downstream effectors. When the Hippo pathway is inactive, YAP is dephosphorylated and translocates into the nucleus, where it binds to transcription factors TEAD1-4 to regulate gene expression (Hansen et al., 2015). YAP has two main isoforms: YAP1 (Szulzewsky et al., 2021) and YAP2 (Oka and Sudol, 2009), with YAP1 being the predominant form. YAP1 is expressed in various cell types and, through TEAD binding, regulates the expression of genes involved in cell proliferation, apoptosis, and stem cell self-renewal (Driskill and Pan, 2023). The downstream target genes regulated by YAP include those associated with cell proliferation, such as *Ctgf* (Moon et al., 2020), *CYR61* (Zhang C. et al., 2024), *C-MYC* (Xiao et al., 2013), *ANKRD1* (Wu et al., 2023), and *AXL* (Okamoto et al., 2023); and apoptosis inhibitors, such as *MCL1* (Glinkina et al., 2023), *BIRC5* (Edwards et al., 2023) and *BIRC2* (Wang Y. et al., 2022). Like YAP1, YAP2 can bind TEAD transcription factors, but its specific functions and regulatory mechanisms are not completely understood (Khanal et al., 2018). Besides TEADs, YAP also interacts with runt-related transcription factor 2 (RUNX2) (Chuang and Ito, 2021), T-box transcription factor 5 (TBX5) (Rosenbluh et al., 2012), and SMADs (Liu H. et al., 2023), promoting their downstream gene transcription, playing a role in cell proliferation, tissue development, anti-apoptosis, and tumorigenesis. Various upstream signals, such as cell polarity complexes, connexins, mechanical cues, cell density, G protein-coupled receptors (GPCRs), soluble factors, receptor tyrosine kinases (RTKs), tight junctions, adherens junctions, integrins, and hypoxic stress, can regulate the Hippo pathway (Fu et al., 2022).

### 2.2 Anatomy and growth of lens epithelial cells

**FIGURE 3:**
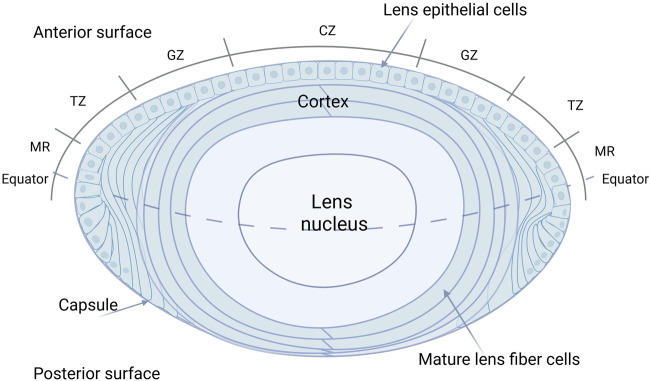
Detailed anatomy of the human eye lens. The LECs located at the anterior side of the equatorial plate proliferate and migrate towards the equatorial region, differentiating into secondary LFCs. The proliferation of LECs occurs in the region above the lens equator, known as the Germination Zone (GZ). The progeny of dividing cells migrate or are displaced from the lens equator below the equator into the Transition Zone (TZ), where they differentiate into fiber cells. During terminal differentiation, LECs exit the cell cycle and degrade subcellular organelles, including the nucleus, ultimately forming mature LFCs with the organelle-free zone (OFZ). CZ: Center zone; GZ: Germinative zone; TZ: Transition zone; MR: Meridional rows.

During embryonic development, the lens placode, derived from the surface ectoderm, invaginates to form the lens vesicle. The anterior epithelial cells of this vesicle form a single layer of lens epithelium, while the posterior epithelial cells differentiate into elongated primary LFCs (Cvekl and Ashery-Padan, 2014). The LECs located at the anterior side of the equatorial plate proliferate and migrate towards the equatorial region, differentiating into secondary LFCs (Liu Z. et al., 2023) (showed in Figure 3). The proliferation of LECs occurs in the region above the lens equator, known as the Germination Zone (GZ). The progeny of dividing cells migrate or are displaced from the lens equator below the equator into the Transition Zone (TZ), where they differentiate into fiber cells (Dawes et al., 2018). During terminal differentiation, LECs exit the cell cycle and degrade subcellular organelles, including the nucleus, ultimately forming mature LFCs with the organelle-free zone (OFZ) (Khokhar et al., 2017). Any abnormalities in the lens development process can lead to lens opacification (Bell et al., 2020). The continuous growth of the lens throughout life is a result of the persistent proliferation of LECs. To achieve and maintain the appropriate size of the lens throughout growth and aging, a strict regulation of the dynamic balance between lens epithelial cell proliferation and fiber differentiation is essential. LECs play a vital role in maintaining lens homeostasis by regulating nutrient transport (Giannone et al., 2021), metabolic activity (Kubota et al., 2016), and cell proliferation (Liu Z. et al., 2023). Multiple factors can interfere with LECs, leading to cataract formation, including oxidative stress, diabetes-related metabolic changes, aging, UV radiation, and chronic inflammation. These factors disrupt LEC function through mechanisms such as protein aggregation, cellular damage, and altered metabolic activity (Munteanu et al., 2024). What’s more, in the study by Katz et al. (2021), it was found that alterations in fiber cell content and intercellular fiber cell binding are likely key contributors to age-related stiffening of the lens, which suggests that changes in LECs may occur during the process of presbyopia.

### 2.3 The role of the Hippo/YAP pathway in lens epithelial cells

#### 2.3.1 The role of Hippo/YAP in ocular diseases

YAP is widely distributed across eye tissues, including the cornea, lens, and retina (Kim et al., 2016). The Hippo pathway is involved in the developmental regulation of ocular tissues, which plays an indispensable role in regulating retinal generation (Yang et al., 2022), retinal neurogenesis (Lee et al., 2018), retinal angiogenesis (Zhu et al., 2018), lens epithelial cell proliferation (Dawes et al., 2018; Kumar et al., 2019) and differentiation, and corneal wound healing (Li et al., 2021). Alterations in the Hippo pathway are complex in ocular diseases, particularly in retina-associated conditions. Angiogenesis accompanied by endothelial cell proliferation is a symptom of diabetic retinopathy (DR). In the retinas of mice with diabetic retinopathy, LATS and TAZ levels are increased, while p-MST and p-YAP are significantly decreased, which indicates that the Hippo pathway plays a role in DR (Hao et al., 2017). Furthermore, a Tyr421His mutation in *Tead1* has been found in patients with Sveinsson chorioretinal atrophy, and this mutant has been shown to disrupt its interaction with *Yap65*, a key component of the Hippo signaling pathway (Fossdal et al., 2004). *MST2* contributes to photoreceptor cell death after retinal detachment, and its absence can protect against this cell death (Matsumoto et al., 2014).

#### 2.3.2 The role of Hippo/YAP in lens epithelial cell proliferation, cell function and differentiation

The role of YAP in the proliferation and differentiation of LECs, maintenance of cell and nuclear morphology, cell polarity, apical polarity complex, connexin morphology, and orderly arrangement of LFCs has been demonstrated in several animal studies (Dawes et al., 2018; He et al., 2019; Kumar et al., 2019; Lu et al., 2020; Li et al., 2023; Wiley et al., 2010). The distribution and nucleocytoplasmic translocation of YAP is associated with its activity state in different regions of the lens. YAP protein is highly distributed in LECs but gradually decreases in the TZ and is almost absent in LFCs. In the GZ, YAP is primarily located in the nucleus and is in a non-phosphorylated, active state, whereas in the TZ, YAP is mainly in the cytoplasm and in a phosphorylated, inactive state (Dawes et al., 2018; Song et al., 2014).

##### 2.3.2.1 YAP regulation of lens epithelial cells proliferation and apoptosis

Research by Song et al. (2014) demonstrated that specific knockout of the *Yap1* gene in developing mice inhibits proliferation and promotes apoptosis of LECs. This study utilized Nestin-Cre to conditionally delete *Yap1*, and the increased apoptosis may be associated with Cleaved Caspase-3. He et al. (2019) discovered that conditional knockout (CKO) of *Yap1* in mice results in cataracts, with a reduction of epithelial cells in the TZ and posterior pole; however, it remains unclear whether apoptosis is significantly affected. This study employed GFAP-Cre to delete *Yap1* in specific cell populations, which may explain the differences in apoptosis observations compared to other studies. Lu et al. (2020) observed that heterozygous deletion of *Yap1* in mice (*Yap1*+/−) leads to reduced proliferation and cell density in LECs. Moreover, overexpression of the *Yap1* target gene *Crim1* can restore the *in vitro* proliferation of *Yap1*+/− LECs, indicating that *Crim1* may be a downstream effector of YAP in promoting LEC proliferation. Kumar et al. (2019) found that the Hippo/Yap pathway responds to upstream mechanical strain signals to regulate LEC proliferation. Using an *ex vivo* stretching device, they demonstrated that in porcine lenses under static stretching, the proliferation level of LECs increases with the amplitude and frequency of stretching. Furthermore, the YAP inhibitor Verteporfin can block the proliferation-promoting effect of static stretching on these cells. These differences in experimental models and Cre lines highlight the complexity of YAP function in LECs and may explain the variability in results across studies.

##### 2.3.2.2 YAP regulation of lens epithelial cell cycle exit and fiber differentiation

Wiley et al. (2010) found that the LFCs in mice with specific deletion of the upstream Hippo pathway gene *Nf2* fail to exit the cell cycle, expressing both fiber cell marker Prox1 and epithelial cell markers FoxE3 and E-cadherin. In *Nf2* CKO mice, the lens vesicle fails to separate from the surface ectoderm, forming multilayered cell masses of lens and corneal epithelial cells on the eye surface, suggesting involvement of the Hippo/YAP pathway in embryonic lens development. YAP integrates cell density information, to transition LECs from a proliferative to a differentiated state, and its absence disrupts the elongation and arrangement of LFCs (Wiley et al., 2010). Fibroblast growth factor (FGF) regulates proliferation and differentiation of LECs in a dose-dependent manner, with low doses inducing proliferation and high doses inducing differentiation to fiber cells (Dawes et al., 2018). Dawes et al. (2018) identified YAP as a downstream effector in FGF-mediated regulation, where low FGF concentrations promote cell proliferation and YAP nuclear translocation, while high concentrations lead to YAP phosphorylation and cytoplasmic translocation, resulting in cell elongation and the expression of fiber markers PROX1 and β-crystallin. Verteporfin can suppress FGF-induced elongation during proliferation and differentiation but does not affect β-crystallin expression in fiber differentiation (Lu et al., 2020).

##### 2.3.2.3 YAP in maintaining morphology and polarity of lens epithelial cells

YAP plays an indispensable role in maintaining the morphology and polarity of lens epithelial cells by regulating cell proliferation, differentiation, and the stability of cell polarity complexes. In YAP-deficient LECs, the morphology changes from cuboidal to nearly flattened, and the localization of polarity markers is significantly altered, leading to reduced intercellular adhesion and disorganization of cellular structures (He et al., 2019; Wiley et al., 2010). Knockout of *Yap1* in LECs leads to changes in cellular and nuclear morphology. In *Yap1* CKO LECs, the localization of the apical polarity complex—including Crumbs, Pals1, Par3, and ZO-1—is disrupted (Song et al., 2014).

### 2.4 Hippo/YAP pathway and age-related cataracts

He et al. (2019) were the first to link YAP with age-related cataract (ARC). Histologically, lenses with *Yap1* knockout show decreased lens epithelial cell density in the TZ and posterior pole, nuclear retention in LFCs, and an accumulation of Morgagnian globules, which are spherical droplets of protein that form in the lens due to the breakdown of cortical cell walls and are often associated with mature Morgagnian cataracts (Deshmukh et al., 2019). Lu et al. (2020) found that heterozygous deficiency of *YAP1* in mice results in cataracts and an abnormal lens epithelial cell phenotype in adulthood. Although early ocular development, including the lens, appears normal, the cataract phenotype of Yap heterozygous mutant animals appears as early as 5 weeks (up to 40% of animals). Liu et al. (2022) were the first to explore the expression levels of YAP in LECs of clinical ARC patients, investigating its role in the pathogenesis of ARC. Increased levels of MST2 and phosphorylated YAP (p-YAP) proteins were observed in LECs from ARC patients and aged mice, along with reduced YAP1 and glucose transporter type 1 (GLUT1) protein expression. Additionally, H_2_O_2_-induced apoptosis in LECs was associated with increased MST2 and p-YAP expression. MST2 overexpression inhibited lens epithelial cell proliferation and promoted apoptosis. Conversely, *YAP1* overexpression enhanced cell proliferation and reduced H_2_O_2_-induced apoptosis. *GLUT1* has been identified as a downstream target of YAP1-TEAD1 co-transcriptional regulation (Liu et al., 2022).

Elevated MST2 expression in ARC patients has been detected, and animal studies report that MST2 activation is related to mitochondrial dysfunction, which induces apoptosis through mitochondrial depolarization (Rauch et al., 2016). YAP is a transcriptional regulator of key mitochondrial autophagy proteins PTEN-induced putative kinase 1/*Pink1* and the E3 ubiquitin ligase Parkin/*Park2* (Jin et al., 2023). In H_2_O_2_-induced oxidative stress in LECs, increased expression of Parkin and mitochondrial autophagy were observed. Overexpression of *PARK2* can promote mitochondrial autophagy, while LECs with low Parkin expression exhibit higher apoptosis levels (Kantorow et al., 2015). YAP’s role in upregulating *PINK1* and Parkin to enhance mitochondrial autophagy in response to environmental stress has been confirmed in non-ocular tissues (Jin et al., 2023; Cho et al., 2021; Xiao et al., 2020; Zhu et al., 2022). In myocardial cells under endotoxin exposure, the absence of MST1, a key upstream component in the Hippo pathway, can activate Parkin-related mitochondrial autophagy, thus reducing mitochondrial damage (Shang et al., 2020). In studies of mitochondrial autophagy in other ocular tissues, inhibition of the *PINK1*-mediated mitochondrial autophagy pathway accelerates microglial aging, leading to retinal ganglion cell damage (Wei et al., 2023). MST2 and MST1 are homologous proteins with overlapping functions; however, MST2 has been more extensively studied in ocular tissues. Further investigation is needed to determine whether the MST2-YAP axis contributes to lens epithelial cell apoptosis and ARC pathogenesis through the transcriptional regulation of key mitochondrial autophagy proteins.

### 2.5 Hippo/YAP pathway and posterior capsule opacification

Phacoemulsification, a surgical technique that uses ultrasound waves to break up the cloudy lens of the eye, combined with intraocular lens implantation is a common clinical treatment for cataract patients. Posterior capsule opacification (PCO) is a frequent secondary complication in patients with intraocular lenses, associated with the plasticity of residual LECs and epithelial-mesenchymal transition (EMT) changes (Wormstone and Eldred, 2016; Tassignon, 2020). In lens epithelial cultures and explants, the knockdown of *Calponin-3* enhances YAP/TAZ transcriptional activity, leading to increased actin stress fiber reorganization and EMT (Maddala et al., 2020). Furthermore, LECs secrete extracellular HSP90-alpha (eHSP90), which promotes the elongation of these cells and upregulates the fibroblast transcription factor PROX1, along with its downstream targets, including LRP1 and AKT. This upregulation of Prox1 is mediated by eHSP90 binding to LRP1, activating the LRP1-AKT pathway, which in turn influences YAP degradation. The interaction between these molecular players suggests that eHSP90-induced Prox1 upregulation significantly contributes to the pathogenesis of PCO by facilitating the differentiation of LECs into fibroblast-like cells (Li et al., 2023). Taiyab A et al. collected anterior capsule samples from three patients with subcapsular cataracts (mean age: 55.3 ± 7.57) and found increased nuclear YAP expression in fibrotic lens capsules through immunohistochemical analysis (Taiyab et al., 2023). And they found YAP co-localized with α-SMA in fibrotic patches in TGFβ-overexpressing mice, unlike in wild-type lenses. Treatment of rat lens explants with the YAP inhibitor verteporfin prevented TGFβ-induced fibrosis, α-SMA and fibronectin expression, and the displacement of E-cadherin and β-catenin. Additionally, LECs co-incubated with TGFβ and the YAP inhibitor did not show increased matrix metalloproteinase 2 induction compared to those treated with TGFβ alone. Therefore, therapeutic strategies aimed at modulating the Hippo/YAP pathway—either by inhibiting YAP activity or activating the Hippo pathway—could be beneficial in improving PCO outcomes.

### 2.6 Hippo/YAP pathway and presbyopia

Presbyopia is an age-related visual impairment where, despite optimal distance correction, near vision clarity is insufficient for an individual’s needs (Wolffsohn and Davies, 2019). With the global population aging, the prevalence of presbyopia is increasing; in 2015, out of an estimated 1.8 billion individuals with functional presbyopia, 826 million had uncorrected near vision impairment (Fricke et al., 2018). The pathogenesis of presbyopia is not yet fully understood, but the reduction or loss of accommodative ability in presbyopic patients may be related to factors such as increased lens volume, increased lens rigidity, and reduced contractile range of the ciliary muscle (Rich and Reilly, 2023; Katz et al., 2021). Lens volume growth results from continuous proliferation and differentiation of LECs, which are crucial in the development of presbyopia.

In the absence of accommodation, the ciliary muscle relaxes, and zonular fibers apply tension to the lens capsule; during accommodation, the tension that flattens the lens is released through ciliary muscle contraction, allowing the lens to elastically return to a more rounded shape. The ciliary muscle remains active into old age (Strenk et al., 1999), and the mechanical properties of the zonular fibers are age-independent (Michael et al., 2012), suggesting that even after presbyopia onset, the human lens capsule might experience periodic tension. The human lens grows continuously throughout life, exhibiting a distinct biphasic growth pattern. Rapid embryonic growth may be driven by a rapid increase in the surface area of the lens capsule and constant tension, whereas postnatal slow growth is delayed due to partial relief of tension during accommodation (Kumar et al., 2019). Lenses of non-accommodating species tend to be larger than those of accommodating species at the same age; for example, a six-month-old pig lens may weigh approximately 400 mg, while a human infant lens weighs around 150 mg (Kumar et al., 2019).

The role of YAP in promoting LEC proliferation has been confirmed by several animal studies. Research by Kumar et al. (2019) was the first to establish a connection between mechanical stretching and the YAP pathway in the lens. Understanding this mechanism is essential for elucidating lens growth, morphogenesis, and potential ways to regulate LEC proliferation. What’s more, Vijay et al. found that the expression of YAP/TAZ and ECM-related genes in human trabecular meshwork cells is influenced by physiologically relevant substrates. YAP was upregulated on softer substrates and downregulated to varying degrees on stiffer ones. Stiffer substrates led to the upregulation of canonical Wnt modulators, TAZ and sFRP-1, which may influence glaucoma progression (Raghunathan et al., 2013). Besides the Hippo signaling pathway, the RHO (Xie et al., 2023) and MAPK/ERK (Qin et al., 2019) signaling pathways are also associated with the mechanical regulation of YAP. Additionally, Wnt, TGF-β, and Notch signaling pathways have been shown to increase cell proliferation in response to shear stress without YAP mediation (Abuammah et al., 2018). Other mechanical cues, such as cell shape, surface tension, and apical pressure, also influence cell proliferation. Further investigation into the role of mechanical signals in LEC proliferation and presbyopia pathogenesis is warranted.

## 3 Conclusion

The Hippo/YAP pathway is integral to the regulation of LECs, influencing critical physiological processes such as proliferation, differentiation, and the maintenance of cellular morphology and polarity. Dysregulation of this pathway has been linked to various lens-related diseases, particularly cataracts and PCO. Recent studies have highlighted the essential role of YAP in the transition of LECs to LFCs, with its activity modulated by mechanical cues and the stiffness of the extracellular matrix. For instance, mechanical strain has been shown to enhance LEC proliferation through YAP activation, indicating a complex interplay between biomechanical factors and cellular signaling pathways (Kumar et al., 2019). Furthermore, the relationship between YAP signaling and oxidative stress suggests that YAP may play a significant role in the pathogenesis of age-related cataracts, as oxidative damage can disrupt normal cellular functions and promote lens opacification (Hong et al., 2024). Future research should aim to elucidate the mechanotransduction pathways that regulate YAP activity in LECs, as understanding these mechanisms could provide insights into lens growth and the development of presbyopia. Additionally, investigating the role of mitochondrial function in lens opacification may reveal novel therapeutic targets, given the importance of mitochondrial health in maintaining cellular homeostasis. Developing targeted therapies that modulate the Hippo/YAP pathway could offer new strategies for preventing or reversing cataract formation. Moreover, correlating findings from animal models with clinical data will be crucial for enhancing our understanding of the pathogenesis of lens diseases and improving diagnostic and therapeutic approaches.

## References

[B1] Abuammah A. Maimari N. Towhidi L. Frueh J. Chooi K. Y. Warboys C. (2018). New developments in mechanotransduction: cross talk of the Wnt, TGF-β and Notch signalling pathways in reaction to shear stress. Curr. Opin. Biomed. Eng. 5, 96–104. 10.1016/j.cobme.2018.03.003

[B2] Bell S. J. Oluonye N. Harding P. Moosajee M. (2020). Congenital cataract: a guide to genetic and clinical management. Ther. Adv. Rare Dis. 1, 2633004020938061. 10.1177/2633004020938061 37180497 PMC10032449

[B3] Bitard J. Grellier E.-K. Lourdel S. Filipe H. P. Hamon A. Fenaille F. (2024). Uveitic glaucoma-like features in Yap conditional knockout mice. Cell Death Discov. 10 (1), 48. 10.1038/s41420-023-01791-6 38272861 PMC10811226

[B4] Cho Y. K. Son Y. Saha A. Kim D. Choi C. Kim M. (2021). STK3/STK4 signalling in adipocytes regulates mitophagy and energy expenditure. Nat. Metab. 3 (3), 428–441. 10.1038/s42255-021-00362-2 33758424

[B5] Chuang L. S. H. Ito Y. (2021). The multiple interactions of RUNX with the hippo–YAP pathway. Cells 10 (11), 2925. 10.3390/cells10112925 34831147 PMC8616315

[B6] Cvekl A. Ashery-Padan R. (2014). The cellular and molecular mechanisms of vertebrate lens development. Development 141 (23), 4432–4447. 10.1242/dev.107953 25406393 PMC4302924

[B7] Dawes L. J. Shelley E. J. McAvoy J. W. Lovicu F. J. (2018). A role for Hippo/YAP-signaling in FGF-induced lens epithelial cell proliferation and fibre differentiation. Exp. Eye Res. 169, 122–133. 10.1016/j.exer.2018.01.014 29355736

[B8] Deshmukh S. Bhattacharjee H. Gupta K. (2019). Triangle sign in Morgagnian cataract. Indian J. Ophthalmol. 67 (1), 137. 10.4103/ijo.IJO_940_18 PMC6324124 30574920

[B9] Driskill J. H. Pan D. (2023). Control of stem cell renewal and fate by YAP and TAZ. Nat. Rev. Mol. Cell Biol. 24 (12), 895–911. 10.1038/s41580-023-00644-5 37626124

[B10] Edwards A. C. Stalnecker C. A. Jean Morales A. Taylor K. E. Klomp J. E. Klomp J. A. (2023). TEAD inhibition overcomes YAP1/TAZ-driven primary and acquired resistance to KRASG12C inhibitors. Cancer Res. 83 (24), 4112–4129. 10.1158/0008-5472.CAN-23-2994 37934103 PMC10821578

[B11] Fossdal R. Jonasson F. Kristjansdottir G. T. Kong A. Stefansson H. Gosh S. (2004). A novel TEAD1 mutation is the causative allele in Sveinsson's chorioretinal atrophy (helicoid peripapillary chorioretinal degeneration). Hum. Mol. Genet. 13 (9), 975–981. 10.1093/hmg/ddh106 15016762

[B12] Fricke T. R. Tahhan N. Resnikoff S. Papas E. Burnett A. Ho S. M. (2018). Global prevalence of presbyopia and vision impairment from uncorrected presbyopia: systematic review, meta-analysis, and modelling. Ophthalmology 125 (10), 1492–1499. 10.1016/j.ophtha.2018.04.013 29753495

[B13] Fu M. Hu Y. Lan T. Guan K. L. Luo T. Luo M. (2022). The Hippo signalling pathway and its implications in human health and diseases. Signal Transduct. Target Ther. 7 (1), 376. 10.1038/s41392-022-01191-9 36347846 PMC9643504

[B14] Giannone A. A. Li L. Sellitto C. White T. W. (2021). Physiological mechanisms regulating lens transport. Front. Physiology 12, 818649. 10.3389/fphys.2021.818649 PMC8735835 35002784

[B15] Glinkina K. A. Teunisse AFAS Gelmi M. C. de Vries J. Jager M. J. Jochemsen A. G. (2023). Combined Mcl-1 and YAP1/TAZ inhibition for treatment of metastatic uveal melanoma. Melanoma Res. 33 (5), 345–356. 10.1097/CMR.0000000000000911 37467061 PMC10470438

[B16] Hansen C. G. Moroishi T. Guan K. L. (2015). YAP and TAZ: a nexus for Hippo signaling and beyond. Trends Cell Biol. 25 (9), 499–513. 10.1016/j.tcb.2015.05.002 26045258 PMC4554827

[B17] Hao G. M. Lv T. T. Wu Y. Wang H. L. Xing W. Wang Y. (2017). The Hippo signaling pathway: a potential therapeutic target is reversed by a Chinese patent drug in rats with diabetic retinopathy. BMC Complement. Altern. Med. 17 (1), 187. 10.1186/s12906-017-1678-3 28372586 PMC5379696

[B18] He Q. Gao Y. Wang T. Zhou L. Zhou W. Yuan Z. (2019). Deficiency of yes-associated protein induces cataract in mice. Aging Dis. 10 (2), 293–306. 10.14336/AD.2018.0910 31011480 PMC6457047

[B19] Hong Y. Sun Y. Ainiwaer M. Xiao B. Zhang S. Ning L. (2024). A role for YAP/FOXM1/Nrf2 axis in oxidative stress and apoptosis of cataract induced by UVB irradiation. FASEB J. 38 (14), e23832. 10.1096/fj.202400848R 39046354

[B20] Jin S. Lee C. J. Lim G. Park S. Lee S. H. Chung J. H. (2023). C-reactive protein accelerates DRP1-mediated mitochondrial fission by modulating ERK1/2-YAP signaling in cardiomyocytes. BMB Rep. 56, 663–668. 10.5483/BMBRep.2023-0127 37817437 PMC10761750

[B21] Kantorow M. Aktan K. Chauss D. Brennan L. A. (2015). Parkin-directed mitophagy is required for lens cell survival upon exposure to cataract-associated environmental insults. Investigative Ophthalmol. and Vis. Sci. 56 (7), 2654.

[B22] Katz J. A. Karpecki P. M. Dorca A. Chiva-Razavi S. Floyd H. Barnes E. (2021). Presbyopia - a review of current treatment options and emerging therapies. Clin. Ophthalmol. 15, 2167–2178. 10.2147/OPTH.S259011 34079215 PMC8163965

[B23] Khanal P. Jia Z. Yang X. (2018). Cysteine residues are essential for dimerization of Hippo pathway components YAP2L and TAZ. Sci. Rep. 8 (1), 3485. 10.1038/s41598-018-21828-6 29472569 PMC5823869

[B24] Khokhar S. K. Pillay G. Dhull C. Agarwal E. Mahabir M. Aggarwal P. (2017). Pediatric cataract. Indian J. Ophthalmol. 65 (12), 1340–1349. 10.4103/ijo.IJO_1023_17 29208814 PMC5742962

[B25] Kim J. Y. Park R. Lee J. H. Shin J. Nickas J. Kim S. (2016). Yap is essential for retinal progenitor cell cycle progression and RPE cell fate acquisition in the developing mouse eye. Dev. Biol. 419 (2), 336–347. 10.1016/j.ydbio.2016.09.001 27616714 PMC5125893

[B26] Kubota M. Shui Y. B. Liu M. Bai F. Huang A. J. Ma N. (2016). Mitochondrial oxygen metabolism in primary human lens epithelial cells: association with age, diabetes and glaucoma. Free Radic. Biol. Med. 97, 513–519. 10.1016/j.freeradbiomed.2016.07.016 27445101 PMC4996752

[B27] Kumar B. Chandler H. L. Plageman T. Reilly M. A. (2019). Lens stretching modulates lens epithelial cell proliferation via YAP regulation. Invest. Ophthalmol. Vis. Sci. 60 (12), 3920–3929. 10.1167/iovs.19-26893 31546253 PMC7043215

[B28] Lee M. Goraya N. Kim S. Cho S.-H. (2018). Hippo-yap signaling in ocular development and disease. Dev. Dyn. 247 (6), 794–806. 10.1002/dvdy.24628 29532607 PMC5980750

[B29] Li H. Li Q. Dang K. Ma S. Cotton J. L. Yang S. (2019). YAP/TAZ activation drives uveal melanoma initiation and progression. Cell Rep. 29 (10), 3200–3211. 10.1016/j.celrep.2019.03.021 31801083 PMC7871510

[B30] Li J. Yu J. Huang W. Sang F. Li J. Ren Y. (2023). Extracellular HSP90 promotes differentiation of lens epithelial cells to fiber cells by activating LRP1‐YAP‐PROX1 axis. FASEB J. 37 (2), e22783. 10.1096/fj.202201187RR 36705056

[B31] Li Y. Ge L. Chen X. Mao Y. Gu X. Ren B. (2021). The common YAP activation mediates corneal epithelial regeneration and repair with different-sized wounds. npj Regen. Med. 6 (1), 16. 10.1038/s41536-021-00126-2 33772031 PMC7997881

[B32] Liu H. Sun M. Wu N. Liu B. Liu Q. Fan X. (2023b). TGF-β/Smads signaling pathway, Hippo-YAP/TAZ signaling pathway, and VEGF: their mechanisms and roles in vascular remodeling related diseases. Immun. Inflamm. Dis. 11 (11), e1060. 10.1002/iid3.1060 38018603 PMC10629241

[B33] Liu S. Su D. Sun Z. Guan L. Wang Z. Zhang G. (2022). High MST2 expression regulates lens epithelial cell apoptosis in age-related cataracts through YAP1 targeting GLUT1. Arch. Biochem. Biophys. 723, 109255. 10.1016/j.abb.2022.109255 35452623

[B34] Liu Z. Huang S. Zheng Y. Zhou T. Hu L. Xiong L. (2023a). The lens epithelium as a major determinant in the development, maintenance, and regeneration of the crystalline lens. Prog. Retin. Eye Res. 92, 101112. 10.1016/j.preteyeres.2022.101112 36055924

[B35] Lu Q. Zhang Y. Kasetti R. B. Gaddipati S. Cvm N. K. Borchman D. (2020). Heterozygous loss of Yap1 in mice causes progressive cataracts. Invest. Ophthalmol. Vis. Sci. 61 (12), 21. 10.1167/iovs.61.12.21 PMC7585397 33085740

[B36] Ma S. Meng Z. Chen R. Guan K. L. (2019). The Hippo pathway: Biology and pathophysiology. Annu. Rev. Biochem. 88, 577–604. 10.1146/annurev-biochem-013118-111829 30566373

[B37] Maddala R. Mongan M. Xia Y. Rao P. V. (2020). Calponin-3 deficiency augments contractile activity, plasticity, fibrogenic response and Yap/Taz transcriptional activation in lens epithelial cells and explants. Sci. Rep. 10 (1), 1295. 10.1038/s41598-020-58189-y 31992794 PMC6987178

[B38] Matsumoto H. Murakami Y. Kataoka K. Lin H. Connor K. M. Miller J. W. (2014). Mammalian STE20-like kinase 2, not kinase 1, mediates photoreceptor cell death during retinal detachment. Cell Death Dis. 5 (5), e1269. 10.1038/cddis.2014.218 24874741 PMC4047884

[B39] Michael R. Mikielewicz M. Gordillo C. Montenegro G. A. Pinilla Cortés L. Barraquer R. I. (2012). Elastic properties of human lens zonules as a function of age in presbyopes. Invest. Ophthalmol. Vis. Sci. 53 (10), 6109–6114. 10.1167/iovs.11-8702 22850416

[B40] Moon S. Lee S. Caesar J. A. Pruchenko S. Leask A. Knowles J. A. (2020). A CTGF-YAP regulatory pathway is essential for angiogenesis and barriergenesis in the retina. iScience 23 (6), 101184. 10.1016/j.isci.2020.101184 32502964 PMC7270711

[B41] Munteanu M. Mocanu V. Preda A. (2024). “Ophthalmological pathology and medical and surgical management in eye disease cataract,” in Clinical ophthalmology: a guide to diagnosis and treatment. Editor Dumitrache M. (Cham: Springer Nature Switzerland), 225–247.

[B42] Nishina H. (2022). Physiological and pathological roles of the Hippo-YAP/TAZ signaling pathway in liver formation, homeostasis, and tumorigenesis. Cancer Sci. 113 (6), 1900–1908. 10.1111/cas.15352 35349740 PMC9207356

[B43] Oka T. Sudol M. (2009). Nuclear localization and pro-apoptotic signaling of YAP2 require intact PDZ-binding motif. Genes Cells 14 (5), 607–615. 10.1111/j.1365-2443.2009.01292.x 19371381

[B44] Okamoto K. Ando T. Izumi H. Kobayashi S. S. Shintani T. Gutkind J. S. (2023). AXL activates YAP through the EGFR-LATS1/2 axis and confers resistance to EGFR-targeted drugs in head and neck squamous cell carcinoma. Oncogene 42 (39), 2869–2877. 10.1038/s41388-023-02810-7 37591955

[B45] Qin X. Li J. Sun J. Liu L. Chen D. Liu Y. (2019). Low shear stress induces ERK nuclear localization and YAP activation to control the proliferation of breast cancer cells. Biochem. Biophysical Res. Commun. 510 (2), 219–223. 10.1016/j.bbrc.2019.01.065 30685085

[B46] Raghunathan V. K. Morgan J. T. Dreier B. Reilly C. M. Thomasy S. M. Wood J. A. (2013). Role of substratum stiffness in modulating genes associated with extracellular matrix and mechanotransducers YAP and TAZ. Investigative Ophthalmol. and Vis. Sci. 54 (1), 378–386. 10.1167/iovs.12-11007 PMC3594895 23258147

[B47] Rauch J. Vandamme D. Mack B. McCann B. Volinsky N. Blanco A. (2016). Differential localization of A-Raf regulates MST2-mediated apoptosis during epithelial differentiation. Cell Death Differ. 23 (8), 1283–1295. 10.1038/cdd.2016.2 26891695 PMC4947672

[B48] Rich W. Reilly M. A. (2023). A review of lens biomechanical contributions to presbyopia. Curr. Eye Res. 48 (2), 182–194. 10.1080/02713683.2022.2088797 35713207

[B49] Rosenbluh J. Nijhawan D. Cox A. G. Li X. Neal J. T. Schafer E. J. (2012). β-Catenin-driven cancers require a YAP1 transcriptional complex for survival and tumorigenesis. Cell 151 (7), 1457–1473. 10.1016/j.cell.2012.11.026 23245941 PMC3530160

[B50] Shang X. Lin K. Zhang Y. Li M. Xu J. Chen K. (2020). Mst1 deletion reduces septic cardiomyopathy via activating Parkin-related mitophagy. J. Cell Physiol. 235 (1), 317–327. 10.1002/jcp.28971 31215035

[B51] Song J. Y. Park R. Kim J. Y. Hughes L. Lu L. Kim S. (2014). Dual function of Yap in the regulation of lens progenitor cells and cellular polarity. Dev. Biol. 386 (2), 281–290. 10.1016/j.ydbio.2013.12.037 24384391 PMC3985746

[B52] Strenk S. A. Semmlow J. L. Strenk L. M. Munoz P. Gronlund-Jacob J. DeMarco J. K. (1999). Age-related changes in human ciliary muscle and lens: a magnetic resonance imaging study. Invest. Ophthalmol. Vis. Sci. 40 (6), 1162–1169. 10235549

[B53] Szulzewsky F. Holland E. C. Vasioukhin V. (2021). YAP1 and its fusion proteins in cancer initiation, progression and therapeutic resistance. Dev. Biol. 475, 205–221. 10.1016/j.ydbio.2020.12.018 33428889 PMC8107117

[B54] Taiyab A. Belahlou Y. Wong V. Pandi S. Shekhar M. Chidambaranathan G. P. (2023). Understanding the role of yes-associated protein (YAP) signaling in the transformation of lens epithelial cells (EMT) and fibrosis. Biomolecules 13 (12), 1767. 10.3390/biom13121767 38136638 PMC10741558

[B55] Tassignon M. J. (2020). Elimination of posterior capsule opacification. Ophthalmology 127 (4s), S27-S28–s8. 10.1016/j.ophtha.2019.12.029 32200822

[B56] Wang Q. Wu H. Zhang X. (2022a). The lens-corneal separation requires precision control of Hippo-Yap signaling. Investigative Ophthalmol. Vis. Sci. 63 (7), 654.

[B57] Wang Y. Chen H. Yu J. Kang W. To K. F. (2022b). Recent insight into the role and therapeutic potential of YAP/TAZ in gastrointestinal cancers. Biochimica Biophysica Acta (BBA) - Rev. Cancer 1877 (5), 188787. 10.1016/j.bbcan.2022.188787 36041574

[B58] Wei M. Zhang G. Huang Z. Ding X. Sun Q. Zhang Y. (2023). ATP-P2X7R-mediated microglia senescence aggravates retinal ganglion cell injury in chronic ocular hypertension. J. Neuroinflammation 20 (1), 180. 10.1186/s12974-023-02855-1 37525172 PMC10392012

[B59] Wiley L. A. Dattilo L. K. Kang K. B. Giovannini M. Beebe D. C. (2010). The tumor suppressor merlin is required for cell cycle exit, terminal differentiation, and cell polarity in the developing murine lens. Investigative Ophthalmol. and Vis. Sci. 51 (7), 3611–3618. 10.1167/iovs.09-4371 PMC2904013 20181838

[B60] Wolffsohn J. S. Davies L. N. (2019). Presbyopia: effectiveness of correction strategies. Prog. Retin. Eye Res. 68, 124–143. 10.1016/j.preteyeres.2018.09.004 30244049

[B61] Wormstone I. M. Eldred J. A. (2016). Experimental models for posterior capsule opacification research. Exp. Eye Res. 142, 2–12. 10.1016/j.exer.2015.04.021 25939555

[B62] Wu L. Ou Z. Liu P. Zhao C. Tong S. Wang R. (2023). ATXN3 promotes prostate cancer progression by stabilizing YAP. Cell Commun. Signal 21 (1), 152. 10.1186/s12964-023-01073-9 37349820 PMC10286397

[B63] Xiao D. Chang W. Ding W. Wang Y. Fa H. Wang J. (2020). Enhanced mitophagy mediated by the YAP/Parkin pathway protects against DOX-induced cardiotoxicity. Toxicol. Lett. 330, 96–107. 10.1016/j.toxlet.2020.05.015 32434049

[B64] Xiao W. Wang J. Ou C. Zhang Y. Ma L. Weng W. (2013). Mutual interaction between YAP and c-Myc is critical for carcinogenesis in liver cancer. Biochem. Biophys. Res. Commun. 439 (2), 167–172. 10.1016/j.bbrc.2013.08.071 23994632

[B65] Xie N. Xiao C. Shu Q. Cheng B. Wang Z. Xue R. (2023). Cell response to mechanical microenvironment cues via Rho signaling: from mechanobiology to mechanomedicine. Acta Biomater. 159, 1–20. 10.1016/j.actbio.2023.01.039 36717048

[B66] Yan Z. Shi H. Zhu R. Li L. Qin B. Kang L. (2018). Inhibition of YAP ameliorates choroidal neovascularization via inhibiting endothelial cell proliferation. Mol. Vis. 24, 83–93. 29422766 PMC5800432

[B67] Yang Y. Jiang X. Li X. Sun K. Zhu X. Zhou B. (2022). Specific ablation of Hippo signalling component Yap1 in retinal progenitors and Müller cells results in late onset retinal degeneration. J. Cell. Physiology 237 (6), 2673–2689. 10.1002/jcp.30757 35533255

[B68] Zhang C. Wei W. Tu S. Liang B. Li C. Li Y. (2024b). Upregulation of CYR61 by TGF-β and YAP signaling exerts a counter-suppression of hepatocellular carcinoma. J. Biol. Chem. 300 (4), 107208. 10.1016/j.jbc.2024.107208 38521502 PMC11021963

[B69] Zhang W. Li J. (2022). EGF receptor signaling modulates YAP activation and promotes experimental proliferative vitreoretinopathy. Investigative Ophthalmol. and Vis. Sci. 63 (8), 24. 10.1167/iovs.63.8.24 PMC9344224 35895037

[B70] Zhang X. Su D. Wei D. Chen X. Hu Y. Li S. (2024a). Role of MST2/YAP1 signaling pathway in retinal cells apoptosis and diabetic retinopathy. Toxicol. Appl. Pharmacol. 484, 116885. 10.1016/j.taap.2024.116885 38447873

[B71] Zhang Y. Xue C. Cui H. Huang Z. (2015). High expression of TAZ indicates a poor prognosis in retinoblastoma. Diagn Pathol. 10, 187. 10.1186/s13000-015-0415-9 26464030 PMC4605227

[B72] Zhu M. Liu X. Wang Y. Chen L. Wang L. Qin X. (2018). YAP via interacting with STAT3 regulates VEGF-induced angiogenesis in human retinal microvascular endothelial cells. Exp. Cell Res. 373 (1), 155–163. 10.1016/j.yexcr.2018.10.007 30342005

[B73] Zhu P. Chen Y. Wang J. Lin G. Wang R. Que Y. (2022). Receptor-Interacting protein kinase 3 suppresses mitophagy activation via the yes-associated protein/transcription factor EB pathways in septic cardiomyopathy. Front. Cardiovasc Med. 9, 856041. 10.3389/fcvm.2022.856041 35402535 PMC8987354

